# Validation of the multi-dimensional scale of perceived social support (MSPSS) and the relationship between social support, intimate partner violence and antenatal depression in Malawi

**DOI:** 10.1186/1471-244X-14-180

**Published:** 2014-06-17

**Authors:** Robert C Stewart, Eric Umar, Barbara Tomenson, Francis Creed

**Affiliations:** 1 Institute of Brain, Behaviour and Mental Health, University of Manchester, Oxford Road, Manchester, UK; 2 Department of Mental Health, College of Medicine, University of Malawi, Blantyre, Malawi; 3 Department of Community Health, College of Medicine, University of Malawi, Blantyre, Malawi; 4 Biostatistics Unit, Institute of Population Health, University of Manchester, Oxford Road, Manchester, UK

**Keywords:** Social support, MSPSS, Antenatal, Depression, Africa

## Abstract

**Background:**

Lack of social support is an important risk factor for antenatal depression and anxiety in low- and middle-income countries. We translated, adapted and validated the Multi-dimensional Scale of Perceived Social Support (MSPSS) in order to study the relationship between perceived social support, intimate partner violence and antenatal depression in Malawi.

**Methods:**

The MSPSS was translated and adapted into Chichewa and Chiyao. Five hundred and eighty-three women attending an antenatal clinic were administered the MSPSS, depression screening measures, and a risk factor questionnaire including questions about intimate partner violence. A sub-sample of participants (n = 196) were interviewed using the Structured Clinical Interview for DSM-IV to diagnose major depressive episode. Validity of the MSPSS was evaluated by assessment of internal consistency, factor structure, and correlation with Self Reporting Questionnaire (SRQ) score and major depressive episode. We investigated associations between perception of support from different sources (significant other, family, and friends) and major depressive episode, and whether intimate partner violence was a moderator of these associations.

**Results:**

In both Chichewa and Chiyao, the MSPSS had high internal consistency for the full scale and significant other, family, and friends subscales. MSPSS full scale and subscale scores were inversely associated with SRQ score and major depression diagnosis. Using principal components analysis, the MSPSS had the expected 3-factor structure in analysis of the whole sample. On confirmatory factor analysis, goodness–of-fit indices were better for a 3-factor model than for a 2-factor model, and met standard criteria when correlation between items was allowed. Lack of support from a significant other was the only MSPSS subscale that showed a significant association with depression on multivariate analysis, and this association was moderated by experience of intimate partner violence.

**Conclusions:**

The MSPSS is a valid measure of perceived social support in Malawi. Lack of support by a significant other is associated with depression in pregnant women who have experienced intimate partner violence in this setting.

## Background

Depression occurring in the antenatal period is an important health problem globally including in low- and middle-income countries (LMIC) [1]. In a meta-analysis of studies from LMIC, weighted mean prevalence of antenatal depression and other common mental disorders was 15.6% (95% confidence interval, CI: 15.4–15.9) [1]. In LMIC, antenatal depression is a risk factor for low birth weight [2,3], prolonged labour [4], delay in initiating breastfeeding [4], and early cessation of exclusive breastfeeding [5].

Lack of social support is an important risk factor for antenatal depression and anxiety in LMIC alongside obstetric, health and other psychosocial factors including intimate partner violence (IPV) [1,6-10]. Social support is a multi-dimensional concept; a key distinction has been made between the *enacted* support an individual receives (that can be externally observed) and the subjective perception of availability and adequacy of support. It is *perceived* social support that has been most closely associated with mental wellbeing [11].

The Multidimensional Scale of Perceived Social Support (MSPSS) is a brief measure of a respondent’s perception of the support that he/she receives from 3 different sources: a significant other, family, and friends [12]. The MSPSS was originally validated in western populations but has since been validated in a number of non-western settings and LMIC [13-22]. Most of these studies found that the MSPSS showed construct validity with scores correlating with measures of depression and anxiety in the expected direction, and good internal consistency for both the full scale and subscales. Of those studies that used exploratory factor analysis (EFA), most found that scores loaded onto the expected 3 factors [14,15,18,19,21] although Urdu versions loaded onto 2 factors in a study from Hong Kong [15] and onto a single factor in a study from Pakistan [20]. In studies that used confirmatory factor analysis (CFA), goodness-of-fit was better for 3-factor models than 2-factor models [13,16,17,23,24]. The MSPSS has been used as a measure of social support in studies of risk factors for perinatal depression in Turkey [25-27], Pakistan [28] and amongst Pakistani women in the UK [29], although only in the latter study were the three different sources of support included as separate variables in the multivariate analysis.

Social support may directly protect against depression, or it may act by buffering the impact of stressful life events [30,31]. Experience of IPV is a risk factor for depression [32] but its effect may be modified by social support [33-36]. We are unaware of any previous publication that has described the relationship between IPV, different sources of social support and antenatal depression in sub-Saharan Africa.

In this paper, we describe the translation and adaptation of the MSPSS into two local languages (Chichewa and Chiyao) as part of our study of depression amongst women attending an antenatal clinic in a predominantly rural district in Malawi. We previously reported the validation of depression screening questionnaires [37] using the Structured Clinical Interview for DSM-IV (SCID) [38] interview as gold standard. We have reported previously also an association between total MSPSS score and antenatal depression [39]. The first aim of the present paper was to assess the internal consistency, factor structure (using both EFA and CFA) and construct validity of the translated and adapted MSPSS in 2 commonly spoken languages in Malawi. The second aim was to examine whether different sources of perceived social support (significant other, family, and friends) showed differing associations with depression. The third aim was to investigate whether IPV was a moderator of the association between depression and perceived social support.

## Method

The study site was the antenatal clinic at Mangochi District Hospital, Mangochi, Malawi. This government hospital is situated in a predominantly rural district at the southern end of Lake Malawi with a population of approximately 800,000 [40]. Women who were attending for their second or later antenatal visit were recruited. In rural Malawi, 44.9% of women attend 4+ antenatal visits and 49.8% 2-3 antenatal visits [41]. Women who were not fluent in one of the two main languages of the area (Chichewa and Chiyao) were excluded.

A convenience sample of women was recruited from the waiting room of the antenatal clinic. Potential participants were approached before seeing the nurse-midwife by 2 fieldworkers, who approached the next woman in line after having completed the previous interview. The information sheet and consent form were given to the participant to read, or were read out by the fieldworker if the participant was illiterate. Written consent was obtained where possible. If a woman consented but was unable to write, verbal consent was documented by the fieldworker. Interviews were conducted in either Chichewa or Chiyao, depending on the participant’s preference. Participants were asked to complete the MSPSS, the depression screening measures and a risk factor questionnaire. Mid-upper arm circumference (MUAC) was measured. Data were collected also from patient-held health records, with participants’ permission. Participants were given a soft drink and a bar of soap to compensate them for the time involved in their participation. The 2 fieldworkers were trained Chichewa- and Chiyao-speaking female school leavers from the local area.

### Measurement of perceived social support

The Multidimensional Scale of Perceived Social Support (MSPSS) is a brief measure of social support designed to measure the respondent’s perception of the adequacy of the support he/she receives [12]. The original version of the MSPSS is a 12-item scale with 7 possible responses to each statement (scored 0-6) giving a score out of a maximum of 72 with higher score indicating greater perceived social support.

We conducted a rigorous process of translation and modification into Chichewa and Chiyao based on that recommended by Rahman et al. [42]. This methodology has been used successfully in an adaptation of the MSPSS in Pakistan [20] and a validation of a depression screening tool in Malawi [43]. A forward translation of the MSPSS was made by Malawian clinical psychologist (EU) and was discussed with a UK psychiatrist (RS) and 2 Malawian social science graduates. RS advised on the concepts captured by the original English wording of each item to guide the choice of Chichewa/Chiyao expression. The draft consensus translation was then back-translated into English by a trilingual independent non-mental health professional. Further modifications were made on the basis of the back-translation. Nurses from the antenatal clinic were invited to comment upon the MSPSS translation. Further modification was made to the translation based on these comments. The MSPSS was administered to a number of women attending the antenatal clinic. The interviewers noted any items about which the subjects asked for clarification and they discussed problematic items with the women in order to check understanding. Experience from the piloting was discussed and the final version was agreed.

Three modifications were made to improve usability in line with an earlier adaptation of the MSPSS in Uganda [21]. Firstly, to allow the scale to be interviewer administered (necessary in a population with a low literacy level), the items were altered from first person statements (e.g. “There is a special person who is around when I am in need”) to second person questions (e.g. “Is there a special person who is around when you are in need?”) (Table 1). Secondly, the number of response options on the Likert scale was reduced from 7 to 5. This altered the range of scores from 0-72 to 0-48. Thirdly, a visual prompt card was designed to facilitate the participants’ response, using simple representations of facial expressions ranging from a grey “sad” face (“strongly disagree*”*) to a bright “happy” face (“strongly agree*”*).

**Table 1:** Multi-dimensional Scale of Perceived Social Support (MSPSS) items in English, adapted into question format

Item 1.	Is there a special person who is around when you are in need?
Item 2.	Is there a special person with whom you can share your joys and sorrows?
Item 3.	Does your family really try to help you?
Item 4.	Do you get the emotional help and support you need from your family?
Item 5.	Do you have a special person who is a real source of comfort to you?
Item 6.	Do your friends really try to help you?
Item 7.	Can you count on your friends when things go wrong?
Item 8.	Can you talk about your problems with your family?
Item 9.	Do you have friends with whom you can share your joys and sorrows?
Item 10.	Is there a special person in your life who cares about your feelings?
Item 11.	Is your family willing to help you make decisions?
Item 12.	Can you talk about your problems with your friends?

### Measurement of depression

Validated Chichewa and Chiyao versions of two depression screening tools were administered to all participants; the Self Reporting Questionnaire (SRQ) and the Edinburgh Postnatal Depression Scale (EPDS). The Self Reporting Questionnaire (SRQ) consists of 20 questions with yes/no answers exploring symptoms of depression and anxiety, and non-specific somatic symptoms commonly associated with distress [44]. The EPDS is a 10-item depression screening measure that was designed for use in the postnatal period [45]; it has also been successfully used antenatally in a number of studies [46]. In a stratified random subsample (with sampling fraction stratified on the basis of score on the SRQ and EPDS), the Structured Clinical Interview for DSM-IV (SCID) was used to diagnose current major or minor depressive episode. On days when the SCID interviewer was present, all participants scoring SRQ ≥8 and/or EPDS ≥ 9 (high scorers), every other participant scoring SRQ 5-7 or EPDS 7-11 (medium scorers), and every fourth participant scoring SRQ 0-4 or EPDS 0-6 (low scorers) were invited to proceed to the SCID interview. SCID interviews took place in a private room near to the antenatal clinic and were conducted by a trained social science graduate-level interviewer. Validation of the Chichewa depression screening measures against DSM-IV depressive disorder is reported in full elsewhere [37]. Any participant causing immediate clinical concern to the interviewer because of active suicidal ideation or self-neglect was counselled and offered referral to local psychiatric care. The EPDS scores were used when determining sampling for SCID interview but are not otherwise analysed in this paper.

### Measurement of potential covariates

Data were collected on variables considered to be potentially associated with maternal depression: age, approximate gestational age, number of years of education completed, marital status, employment, primigravida, number of own living children, previously experienced the death of one of own children, wealth (measured using the WHO-designed assets questionnaire as used in the Demographic and Health Survey (DHS) national survey of households [47], MUAC, change in maternal weight per day between first antenatal visit and recruitment as recorded in maternal health record (kg/day), HIV status, whether taking antiretroviral treatment (ART), response of the father of the child to the pregnancy, experience of IPV (ascertained by asking whether the participant’s current or most recent partner had ever hit her or forced her to have sex when she did not want to), language in which the interview was conducted, and which of the two fieldworkers conducted the interview.

#### Statistical analysis

Two participants had no data for MSPSS. Missing values for 11 participants who had 1 item missing and 1 participant who had 2 items missing on the MSPSS were imputed using mean substitution for the same subscale and the same participant. Mean (Standard Deviation), median (IQR) and Cronbach’s alpha (a measure of internal consistency) for MSPSS total score and the 3 subscales were calculated for three groups: Chichewa speakers, Chiyao speakers and the whole sample.

Principal Components Analysis was used to explore the factor structure of the MSPSS, at first unrotated and then with promax rotation with the number of components determined by the number of components with eigenvalues > 1.0 in the unrotated matrix. Principal Components Analysis was conducted on the Chichewa speakers, Chiyao speakers and the whole sample. For each language group, we conducted separate analyses grouped by which fieldworker administered the MSPSS.

The 12 items of the MSPSS were subject to CFA using Stata version 13 (StatCorp LP, College Station, Texas). The covariance matrix was analysed using maximum likelihood, and the hypothesised model consists of either 3 first-order factors (family, friends, significant other) or 2 first-order factors (family and significant other combined and friends) and a second order factor (perceived social support). Both 3-factor and 2-factor models were examined in the whole group (n = 583), in the Chichewa subgroup (n = 269) and in the Chiyao subgroup (n = 314). For each model, the following goodness-of fit-statistics are presented: chi-squared, comparative fit index (CFI), standardised root mean square residual (SRMR), root mean squared error of approximation (RMSEA) and Bayesian Information Criteria (BIC). For a model to be considered a good fit, CFI should be at least 0.96, SRMR ≤ 0.06 and RMSEA ≤ 0.05 [48]. Ideally the chi-squared value should be non-significant, but since it is so sensitive to large sample sizes, this is unlikely. Therefore, by convention in CFA, it is used to test the relative fit of different models rather than a test of each. Similarly, BICs are not useful for evaluating a single model. They are only useful for comparing different models on the same set of data; models which fit the data well will have lower scores. Since the initial models did not fit the data very well, in the 3-factor models, modification indices were used to determine pairs of items which were highly correlated and the CFA was repeated allowing correlations between items. A satisfactory fit for CFI was achieved allowing correlations between items 6 and 7, and items 3 and 4, but to achieve a satisfactory fit for RMSEA, it was necessary to allow correlation between successive item pairs 6 & 7, 3 & 4, 1 & 2, 4 & 12, 3 & 12, and 9 & 10.

Univariate analyses of associations between MSPSS total, subscale scores and dependent variables (maternal SRQ score and major depressive episode) were conducted using Spearman’s correlation coefficients and Student’s independent *t*-test respectively. For the analysis of associations with diagnosis of major depressive episode in both univariate and multivariate analyses, inverse probability sampling weights were used to account for the different sampling percentages of high, medium and low SRQ/EPDS scorers. These are the reciprocal of the sampling fraction, and must be used in two-phase studies [49]. This method adjusts both the prevalence and its standard error according to the different sampling fractions.

Using inverse sample weighting to account for different sampling of high, medium and low scores on the SRQ/EPDS, the 3 MSPSS subscales and other variables associated with maternal SRQ at the p < 0.1 level (age, MUAC, being unmarried, having own business, years of schooling, unplanned pregnancy, an unsupportive reaction to the pregnancy from the child’s father, experienced IPV, complications in a previous pregnancy, primigavida, questionnaire language and fieldworker) [39] were entered into a logistic regression with major depressive episode vs. remainder as the dependent variable. Unsupportive reaction to the pregnancy from the child’s father was excluded because of overlap of this variable with MSPSS significant other subscale. Variance Inflation Factors (VIF) of the independent variables entered were checked to ensure that none was above 2, which may cause problems of multi-colinearity. The maximum value for VIF was 1.82. There was only a small amount of missing data, ranging from none for most variables to a maximum of 18 (3% of the women) for MUAC. Stata imputation was used to impute values for missing data on the independent variables, using the remaining variables in the model. This repeated the random imputations for each item of missing data 10 times so that standard errors of all estimated parameters could be adjusted appropriately [50].

In order to test the possibility of the effect of social support on depression being moderated by IPV, the logistic regression analyses were repeated with an additional term for the interaction between IPV and each of the 3 MSPSS subscales in turn. As significant other was the only subscale with a significant interaction term, the logistic regression analyses were repeated again to test the effect of the significant other score for participants who had or had not experienced IPV.

#### Ethical Approval

Ethical Approval for the study was given by the College of Medicine Research Ethics Committee, Malawi (P.06/11/1089).

## Results

### Sample Characteristics

Data collection took place on 38 consecutive clinic days in November 2011 to January 2012. From clinic records, 1431 women attended clinic for second or later antenatal visit on these days. 599 women were invited to participate in the study, of whom 583 (98.9%) were recruited; the remainder refused. Data are presented in this paper for these 583 participants (269 Chichewa speakers, 314 Chiyao speakers). Those recruited were significantly older than those who refused or were not approached (mean age 25.14 (SD 6.22) n = 572 vs 23.81 (SD 5.80) n = 713, t = 3.983 p < 0.001). There was no difference between those recruited or not recruited in gestational age or rate of change in weight from 1^st^ antenatal visit to recruitment antenatal visit. SCID interviews took place on 33 of the 38 days of the study. On days when SCID interviews were done, 503 women were recruited. 196 were interviewed using SCID and this group was included in analyses concerning major depressive episode. The sampling fractions for low, medium and high scorers on the SRQ/EPDS were 87/351, 50/85 and 59/67 respectively [37].

### MSPSS internal consistency, factor structure and correlations with SRQ score and major depressive episode

Mean (SD), median (IQR) and Cronbach’s alpha for MSPSS total score and subscales are shown in Table 2 for Chichewa speakers, Chiyao speakers and the whole sample. It can be seen that the results are almost identical for the Chichewa and Chiyao samples. Cronbach’s alpha was high throughout. The 3 subscales scores were moderately correlated (Spearman correlation coefficients: significant other/family 0.608, significant other/friends 0.501, family/friends 0.627 (all p values < 0.001)).

**Table 2:** Mean (SD), median (interquartile range) and Cronbach’s alpha for MSPSS total and subscales

	**Whole sample**			**Chichewa**			**Chiyao**		
	**Mean (SD)**	**Median (IQR)**	**Cronbach’s alpha (n)**	**Mean (SD)**	**Median (IQR)**	**Cronbach’s alpha (n)**	**Mean (SD)**	**Median (IQR)**	**Cronbach’s alpha (n)**
MSPSS total	39.29	41	0.900	38.89	40	0.881	39.62	41	0.916
	(8.79)	(34-48)	(n = 569)	(8.56)	(33-47.5)	(n = 269)	(8.99)	(35-48)	(n = 308)
MSPSS significant other subscale	14.41	16	0.855	14.48	16	0.854	14.35	16	0.855
	(2.79)	(14-16)	(n = 580)	(2.63)	(14-16)	(n = 267)	(2.93)	(14-16)	(n = 313)
MSPSS family subscale	13.25	15	0.889	12.87	15	0.915	13.58	15	0.849
	(3.63)	(12-16)	(n = 574)	(4.10)	(11-16)	(n = 264)	(3.14)	(12-16)	(n = 310)
MSPSS friends subscale	11.62	13	0.874	11.54	13	0.895	11.69	13	0.854
	(4.33)	(8-16)	(n = 576)	(4.46)	(9-16)	(n = 265)	(4.22)	(8-16)	(n = 311)

(Subjects with MSPSS done, mean substitution of occasional missing items: whole sample (n = 581), Chichewa speakers (n = 268) and Chiyao speakers (n = 313)).

#### Principal components analysis

The pattern matrices of PCA with promax rotation of the MSPSS for the whole sample, Chichewa speakers and Chiyao speakers are shown in Table 3. When the data collected by each fieldworker were analysed separately, for Chichewa speakers the MSPSS resolved to the expected 3 factors for both fieldworkers; for Chiyao speakers the MSPSS resolved to the expected 3 factors for one fieldworker but to only a single factor for the other fieldworker.

**Table 3:** Principal component analysis of MSPSS whole sample, Chichewa and Chiyao speakers

	**Whole sample**			**Chichewa**			**Chiyao**	
	**Component**			**Component**			**Component**	
	**1**	**2**	**3**	**1**	**2**	**3**	**1**	**2**
Item 3 (Family)	-0.027	0.903	-0.025	0.887	-0.027	0.042	0.645	0.145
Item 4 (Family)	-0.082	0.928	0.037	0.924	-0.056	0.046	0.818	0.017
Item 8 (Family)	0.022	0.841	-0.008	0.934	0.029	-0.117	0.731	0.058
Item 11 (Family)	0.113	0.772	0.002	0.816	0.064	0.036	0.543	0.291
Item 6 (Friends)	0.933	-0.012	-0.063	-0.043	0.933	-0.005	-0.097	0.965
Item 7 (Friends)	0.941	0.005	-0.069	-0.011	0.936	-0.039	-0.063	0.957
Item 9 (Friends)	0.629	0.060	0.155	0.074	0.716	0.071	0.395	0.399
Item 12 (Friends)	0.859	-0.035	0.034	-0.001	0.884	-0.007	0.070	0.771
Item 1 (Significant other)	-0.066	0.008	0.888	0.039	-0.061	0.890	0.880	-0.159
Item 2 (Significant other)	-0.082	-0.052	0.923	-0.053	-0.098	0.926	0.838	-0.108
Item 5 (Significant other)	0.114	0.101	0.684	0.057	0.148	0.709	0.766	0.058
Item 10 (Significant other)	0.088	-0.008	0.791	-0.033	0.066	0.806	0.802	0.016

Rotation Method: Promax with Kaiser Normalization. Pattern matrices shown. (Missing cases excluded listwise).

#### Confirmatory factor analysis

In each case the 3-factor model was a better fit than the 2-factor model according to all criteria (Table 4) and also showed a significant reduction in the chi-squared value at p < 0.001. The 3-factor model did not fit quite so well for Chiyao as it did for Chichewa. For the whole group the fit was better if items 3&4 were allowed to be correlated as well as items 6&7. In this final model, CFI > 0.96 although RMSEA remained > 0.05. In this model, factor loadings all exceeded 0.7. For the latent variable, family, the standardised loadings are 0.76 for item 3, 0.82 for item 4, 0.81 for item 8 and 0.82 for item 11. For friends they are 0.74 for item 6, 0.77 for item 7, 0.71 for item 9 and 0.84 for item 12. For significant other they are 0.80 for item 1, 0.78 for item 2, 0.76 for item 5 and 0.75 for item 10.

**Table 4:** Goodness of fit indices for the MSPSS whole sample, Chichewa and Chiyao speakers

	**3-factor model**					**2-factor model**				
	***X***^**2**^**(51)**	**CFI**	**SRMR**	**RMSEA**	**BIC**	***X***^**2**^**(53)**	**CFI**	**SRMR**	**RMSEA**	**BIC**
Without correlated errors										
All (n = 583)	373.4	0.926	0.070	0.104	16,386	859.9	0.816	0.094	0.162	16,860
Chichewa (n = 269)	157.2	0.952	0.070	0.088	7,507	532.8	0.781	0.126	0.183	7,872
Chiyao (n = 314)	349.0	0.875	0.083	0.136	8,833	404.3	0.853	0.084	0.145	8,877
With correlated errors for items 6 and 7										
All (n = 583)	271.6	0.949	0.047	0.087	16,291	757.7	0.839	0.078	0.153	16,764
Chichewa (n = 269)	118.6	0.969	0.059	0.071	7,474	494.2	0.798	0.120	0.178	7,839
Chiyao (n = 314)	275.7	0.906	0.051	0.120	8,766	325.5	0.886	0.054	0.129	8,804
With correlated errors for items 6 and 7, and for items 3 & 4.										
All (n = 583)	219.0	0.961	0.043	0.077	16,244	609.5	0.872	0.070	0.137	16,622
Chichewa (n = 269)	116.7	0.969	0.058	0.072	7,479	488.5	0.800	0.116	0.179	7,838
Chiyao (n = 314)	216.2	0.930	0.046	0.104	8,712	243.8	0.919	0.050	0.110	8,728

CFI = Comparative Fit Index.

SRMR = Standardised Root Mean Square Residual.

RMSEA = Root Mean Squared Error of Approximation.

BIC = Bayesian Information Criteria.

#### Construct validity

SRQ total score was negatively correlated with MSPSS total (Spearman correlation coefficient r_s_ = -0.287, p < 0.001), significant other (r_s_ = -0.210, p < 0.001), family (r_s_ = -0.301, p < 0.001) and friends (r_s_ = -0.191, p < 0.001) subscales. Table 5 shows significant differences in mean (SD) MSPSS and subscale scores for those with and without major depressive episode (Student’s independent *t*-test). These data are for the total sample. When the Chichewa and Chiyao measures were analysed separately, all scales were significantly associated with SRQ score and major depressive episode, except for the friends subscale with major depressive episode in the Chiyao sample (p = 0.15) (data available on request).

**Table 5:** **Adjusted mean (SE) MSPSS scores in major depression vs minor or no depression groups (Student’s independent *t*-test)**

	**Adjusted mean (SE) MSPSS**		
	**Major depression**	**Minor or no depression**	**P value**
MSPSS total	31.6 (2.24)	41.6 (0.58)	<0.001
Significant other subscale	11.6 (0.80)	14.9 (0.15)	<0.001
Family subscale	10.2 (0.86)	13.9 (0.24)	<0.001
Friends subscale	9.8 (0.95)	12.7 (0.32)	0.004

(Sample inverse weighted up to total sample recruited on days when SCID done, n = 503).

### Investigation of the relationship between source of social support, intimate partner violence and antenatal depression

In a logistic regression with major depressive episode as the dependent variable and total MSPSS as an independent variable, the two variables that were significantly associated with major depressive episode were MSPSS total score (OR = 0.90; 95% CI: 0.85 to 0.96) and experience of IPV (OR = 19.0; 95% CI: 5.76 to 62.7). When this analysis was repeated with the 3 subscales of MSPSS entered as independent variables, instead of total MSPSS score, MSPSS significant other subscale score, experience of IPV and higher number of years of schooling were the significant correlates (Table 6).

**Table 6:** Logistic Regression: odds ratio and 95% confidence interval for possible correlates of Major Depressive Episode

	**Odds ratio**	**[95% Confidence interval]**	**P value**
MSPSS significant other subscale score	0.66	0.47 - 0.94	0.020
MSPSS family subscale score	0.89	0.74 - 1.06	0.193
MSPSS friends subscale score	1.05	0.90 - 1.23	0.526
Age	1.06	0.93 - 1.21	0.377
Mid-upper arm circumference	1.26	0.95 - 1.66	0.107
Not married	6.43	0.97 - 42.5	0.053
Conducts own business	0.14	0.01 - 1.51	0.105
Number of years of schooling	1.21	1.01 - 1.45	0.039
Pregnancy unplanned	1.08	0.37 - 3.20	0.887
Experienced intimate partner violence	18.5	4.98 - 68.6	<0.001
Experienced complication in previous pregnancy/delivery	0.20	0.03 - 1.28	0.088
Primigravida	0.43	0.07 - 2.65	0.364
Chiyao questionnaire used	0.97	0.28 - 3.40	0.962
Fieldworker 2	1.52	0.33 - 6.97	0.592

(Sample inverse weighted up to total sample recruited on days when SCID done, n = 503, Stata imputation command used to impute values for missing data on the independent variables).

When the MSPSS family score by IPV interaction term was included in this analysis, it was not a significant correlate of depression (p = 0.57). The same was true of the MSPSS friends score by IPV interaction term (p = 0.90). The MSPSS significant other score by IPV interaction term was a significant predictor (p = 0.048). The logistic regression with major depressive episode as the dependent variable was repeated separately for those with and without IPV (Tables 7 and 8). For the group who did not report IPV, significant other subscale was not significantly associated with major depressive episode (OR = 0.84, 95% CI 0.59 to 1.20, p = 0.35), but for the group who reported IPV, significant other subscale was significant (OR = 0.44, 95% CI 0.25 to 0.75, p = 0.003).

**Table 7:** Logistic Regression in group who had not experienced intimate partner violence: odds ratio and 95% confidence interval for possible correlates of Major Depressive Episode

	**Odds Ratio**	**[95% Confidence interval]**	**P value**
MSPSS significant other subscale score	0.85	0.59 - 1.20	0.349
MSPSS family subscale score	0.83	0.64 - 1.06	0.129
MSPSS friends subscale score	0.99	0.80 - 1.22	0.929
Age	1.01	0.85 - 1.20	0.937
Mid-upper arm circumference	1.44	0.96 - 2.14	0.077
Not married	3.37	0.24 - 47.45	0.365
Number of years of schooling	1.25	0.98 - 1.59	0.076
Pregnancy unplanned	0.89	0.18 - 4.49	0.889
Experienced complication in previous pregnancy/delivery	0.50	0.05 - 5.38	0.566
Primigravida	0.23	0.03 - 2.06	0.188
Chiyao questionnaire used	0.40	0.05 - 3.06	0.372
Fieldworker 2	1.46	0.29 - 7.44	0.645

(Sample inverse weighted up to total sample recruited on days when SCID done, n = 397, Stata imputation command used to impute values for missing data on the independent variables) Note: business has been excluded from this analysis because there were no SCID cases for business women.

**Table 8:** Logistic Regression in group who had experienced intimate partner violence: odds ratio and 95% confidence interval for possible correlates of Major Depressive Episode

	**Odds Ratio**	**[95% Confindence interval]**	**P value**
MSPSS significant other subscale score	0.44	0.26 - 0.75	0.003
MSPSS family subscale score	0.86	0.68 - 1.10	0.235
MSPSS friends subscale score	0.96	0.74 - 1.25	0.761
Age	1.03	0.80 - 1.33	0.820
Mid-upper arm circumference	1.52	1.06 - 2.17	0.024
Not married	2.12	0.11 - 41.83	0.616
Conducts own business	0.82	0.08 - 8.58	0.866
Number of years of schooling	1.31	0.98 - 1.74	0.065
Pregnancy unplanned	3.61	0.44 - 29.40	0.226
Experienced complication in previous pregnancy/delivery	0.06	0.00 - 1.90	0.107
Primigravida	0.74	0.05 - 11.84	0.829
Chiyao questionnaire used	7.12	0.61 - 83.50	0.116
Fieldworker 2	1.95	0.21 - 17.99	0.549

(Sample inverse weighted up to total sample recruited on days when SCID done, n = 106, Stata imputation command used to impute values for missing data on the independent variables).

## Discussion

This is the first study to adapt and validate the MSPSS in Malawi, and the second such study in a rural setting in sub-Saharan Africa. It is the first study to investigate the relationship between source of social support, depression and IPV in an antenatal population in sub-Saharan Africa. We found that the MSPSS could be successfully translated and adapted for use in Malawi with some modifications. The translation and adaptation appeared to be successful in both Chichewa and Chiyao, although the 3-factor model was a better fit in Chichewa than Chiyao. The total scale and subscales showed good internal consistency and demonstrated construct validity in that they were associated with measures of depression in the expected direction. We found that a low score (indicating lower perceived adequacy of support) on the significant other subscale but not the family and friends subscales, was associated with major depressive episode after adjustment for covariates, and that IPV was an effect modifier of this association.

One of the strengths of the study was the rigorous process of translation and adaptation of the MSPSS to ensure semantic equivalence with the original. As Nakigudde et al. [21] in Uganda, we improved local ease of use by incorporating a visual prompt and reducing the number of possible responses for each item, although this has lessened technical equivalence with the original version. A further strength was that we could demonstrate the construct validity of the MSPSS by investigating associations with both a continuous measure of depressive/anxious symptoms (SRQ) and a diagnostic interview (SCID) for major depressive disorder. For the first time, we assessed whether the three subscale scores of MSPSS interacted with IPV in logistic regression.

There were a number of limitations of the study. Firstly, we did not carry out test-retest or inter-rater reliability testing. This is important as we found a difference in the factor analysis results for the two fieldworkers in Chiyao. It is not clear whether this reflects inadequacies of our translation, ambiguities in that language or inadequate training of the fieldworkers. Since a single interviewer conducted the SCID interviews we could not assess inter-rater reliability of this interview; the interviewer was not an experienced mental health clinician but received training and supervision from mental health professionals, a method we have used successfully in the past [43].

Because of the high number of attendees and the busy nature of the clinic we used convenience sampling, which could have introduced selection bias. Women who refused the study were slightly younger than the participants but maternal age was not associated with depression in this study, so it is unlikely that this has seriously affected our results. The response rate for SCID interviews was low amongst women with a low SRQ/EPDS but satisfactory for the other groups. It is possible that we recruited a skewed sample of the women with few psychiatric symptoms and this might have affected our results if those who refused had less social support than those who agreed to be included. Confidence intervals around the odds ratios in the logistic regression are wide reflecting small numbers in some of the groups sampled for SCID interview.

It is difficult to generalize our results to a community population. Although in rural Malawi take-up of antenatal care is high [41], suggesting that the sample is likely to be broadly representative, our district hospital antenatal clinic-based sample may have included more women with previous obstetric complications or problems in the current pregnancy than a health centre clinic. Finally, the cross-sectional design of this study does not allow any conclusions to be drawn regarding the causal direction of any associations, and there is also risk of information bias as women with depression may be more likely to report dissatisfaction with social support because of negative cognitive bias. A prospective study investigating whether social support during pregnancy predicts depression later in the perinatal period, adjusting for baseline depression, would overcome this limitation.

The MSPSS resolved to the expected 3 factors on PCA for the whole sample and Chichewa speakers; this is consistent with most other validation studies in non-western settings and LMIC [13-18,21]. In Chiyao, the MSPSS resolved to a two-factor structure on PCA; further analysis showed that for data collected by one fieldworker the MSPSS had a 1-factor structure and for the other, a 3-factor structure. CFA demonstrated that the 3-factor model was a better fit for the data than a 2-factor model in both languages, although the fit in Chiyao was poorer than in Chichewa. For the whole sample, most of the goodness-of-fit statistics met accepted criteria once correlation between items was allowed. RMSEAs were still not a very good fit in the final 3-factor model that allowed correlation between items 3&4 and 6&7. However, this was consistent with several previous studies in LMIC [13,16,22,24].

As in this study, Tonsing et al. [15] found a different factor structure for two translations of the MSPSS (3 factors in Nepali, 2 factors in Urdu) in a Hong Kong study. Our finding that the 3-factor structure was less robust in Chiyao might reflect true differences in support structures between Chichewa and Chiyao speakers. Alternatively, it may be related to limitations in our adaptation of the MSPSS such that, for one fieldworker, instructions were not sufficiently clear. Thirdly, it might reflect difficulty in questionnaire understanding; Chiyao speakers had significantly fewer years of education than Chichewa speakers (unpublished data).

The relationship of the significant other to the respondent is not specified in the original MSPSS and is termed “a special person” in the questionnaire items [12]. Thus, it could refer to someone who is also regarded by the respondent as belonging to “family” or “friends”. It been suggested that this may explain instances when significant other and family or significant other and friends do not differentiate into separate factors on EFA [15]. In the revised Thai version, Wongaparen et al. [17] added an instruction that told respondents that significant other meant “not family or friends”; this improved the test characteristics. However, this instruction was not in the original version and thus lessens technical equivalence with the original. In further studies it would be useful to record the relationship of the “significant other” for each participant.

Our finding that support from a significant other, rather than support from family or friends, is associated with risk of major depressive episode, and is the only aspect of support to show interaction with IPV, is a particularly interesting result of this study. Firstly, it suggests a true difference between the subscales of the MSPSS. Secondly it provides a more complete picture of the role of social support in protecting against depression in this population of pregnant women in a low-income country. It may be that significant other support was associated with depression because the concept of a “special person” implies an especially close relationship. The wording of the MSPSS items concerning support from a “significant other” (items 1, 2, 5 & 10 in Table 1) emphasize the close confiding nature of this relationship and is consistent with the notion that being able to confide in another is key in protecting against depression [51].

Studies of social support and antenatal depression in high income countries have found that support by an intimate partner, more than social support in general, is strongly associated with freedom from depression [52]. Studies from LMIC have found associations between absence of antenatal depression and both intimate partner and family support [1]. In studies from Africa, antenatal depression has been associated with lack of perceived support from partner [53], and with low level of intimacy with partner [8]. Neither study enquired about support from other sources. Our finding extends this previous evidence, in that we found that support by a significant other was protective against depression only in those who had experienced IPV. This suggests the importance of key supportive relationships outside the marital relationship, although it is not impossible that for some respondents the spouse who perpetrated IPV might also be seen as a source of support. Further investigation is needed to determine this.

Social support may act to directly reduce the risk of depression by promoting positive mood states and improving health-related behaviours, or it may protect against depression only in people under stress, i.e. a buffering effect [30,31,54]. This study supports a buffering effect of perceived significant other support against depression in those exposed to IPV. This is important as IPV is a traumatic life event that was reported by 22% of the pregnant women in our study [39]. We analyzed our data to examine whether IPV moderated the association between social support and major depression as the reported IPV may have occurred at some time in the past whereas the social support and depression measures refer to the present. A number of studies from the USA have found a buffering effect of social support on the impact of IPV on mental health outcomes [33-36]. Our study supports the conclusion that, for those who have experienced IPV, the ability to confide in a close other is crucial in protecting against depression.

## Conclusion

The MSPSS is a valid measure of perceived social support in Malawi that successfully discriminates between sources of support. Perceived social support from a significant other may act as a buffer to the effect of IPV on depression in pregnant women in rural Malawi. There are currently no evidence-based interventions available for the prevention and treatment of perinatal depression in this population. This study suggests that a trial of a psychosocial intervention similar to that which has been developed in Pakistan [55] is warranted and that it should include a focus on the activation of supportive relationships amongst women experiencing IPV.

## Competing interests

The authors declare that they have no competing interests.

## Authors’ contributions

RS lead the design and conduct of the study, data analysis and manuscript drafting. EU was involved in study design, study conduct and manuscript drafting. FC supervised the project and contributed to project design, analysis and manuscript drafting. BT advised on analysis and interpretation of the data. All authors read and approved the final manuscript.

## Pre-publication history

The pre-publication history for this paper can be accessed here:

http://www.biomedcentral.com/1471-244X/14/180/prepub

## References

[B1] Fisher J Cabral De Mello M Patel V Rahman A Tran T Holton S Holmes W Prevalence and determinants of common perinatal mental disorders in women in low- and lower-middle-income countries: a systematic review Bull World Health Organ 2012 90 139G 149G 10.2471/BLT.11.091850 PMC3302553 22423165

[B2] Patel V Prince M Maternal psychological morbidity and low birth weight in India Br J Psychiatry 2006 188 284 285 16507972 10.1192/bjp.bp.105.012096

[B3] Rahman A Iqbal Z Bunn J Lovel H Harrington R Impact of maternal depression on infant nutritional status and illness: a cohort study Arch Gen Psychiatry 2004 61 946 15351773 10.1001/archpsyc.61.9.946

[B4] Hanlon CA Medhin G Alem A Tesfaye F Lakew Z Worku B Dewey M Araya M Abdulahi A Hughes M Tomlinson M Patel V Prince M Impact of antenatal common mental disorders upon perinatal outcomes in Ethiopia: the P-MaMiE population-based cohort study Trop Med Int Health 2009 14 156 166 19187514 10.1111/j.1365-3156.2008.02198.x

[B5] Patel V Rodrigues M DeSouza N Gender, poverty, and postnatal depression: a study of mothers in Goa, India Am J Psychiatry 2002 159 43 47 11772688 10.1176/appi.ajp.159.1.43

[B6] Abiodun OA Adetoro OO Ogunbode OO Psychiatric morbidity in a pregnant population in Nigeria Gen Hosp Psychiatry 1993 15 125 128 8472940 10.1016/0163-8343(93)90109-2

[B7] Adewuya AO Ola BA Aloba OO Mapayi BM Anxiety disorders among Nigerian women in late pregnancy: a controlled study Arch Womens Ment Health 2006 9 325 328 17033737 10.1007/s00737-006-0157-5

[B8] Esimai OA Fatoye FO Quiah AG Vidal OE Momoh RM Antepartum anxiety and depressive symptoms: a study of Nigerian women during the three trimesters of pregnancy J Obstet Gynaecol 2008 28 202 203 18393020 10.1080/01443610801912352

[B9] Hartley M Tomlinson M Greco E Comulada WS Stewart J le Roux I Mbewu N Rotheram-Borus MJ Depressed mood in pregnancy: prevalence and correlates in two Cape Town peri-urban settlements Reprod Health 2011 8 9 21535876 10.1186/1742-4755-8-9 PMC3113332

[B10] Dibaba Y Fantahun M Hindin MJ The association of unwanted pregnancy and social support with depressive symptoms in pregnancy: evidence from rural Southwestern Ethiopia BMC Pregnancy Childbirth 2013 13 135 23800160 10.1186/1471-2393-13-135 PMC3716614

[B11] Haber MG Cohen JL Lucas T Baltes BB The relationship between self-reported received and perceived social support: a meta-analytic review Am J Community Psychol 2007 39 133 144 17308966 10.1007/s10464-007-9100-9

[B12] Zimet GD Dahlem NW Zimet SG Farley GK The multidimensional scale of perceived social support J Pers Assess 1988 52 30 41 10.1080/00223891.1990.9674095 2280326

[B13] Bruwer B Emsley R Kidd M Lochner C Seedat S Psychometric properties of the multidimensional scale of perceived social support in youth Compr Psychiatry 2008 49 195 201 18243894 10.1016/j.comppsych.2007.09.002

[B14] Eker D Arkar H Perceived social support: psychometric properties of the MSPSS in normal and pathological groups in a developing country Soc Psychiatry Psychiatr Epidemiol 2010 30 121 126 7624805 10.1007/BF00802040

[B15] Tonsing K Zimet GD Tse S Assessing social support among South Asians: the multidimensional scale of perceived social support Asian J Psychiatr 2012 5 164 168 22813661 10.1016/j.ajp.2012.02.012

[B16] Wongpakaran T Wongpakaran N Ruktrakul R Reliability and validity of the multidimensional scale of perceived social support (MSPSS): Thai Version Clin Pract Epidemiol Ment Health 2011 7 161 166 22114620 10.2174/1745017901107010161 PMC3219878

[B17] Wongpakaran N Wongpakaran T A revised thai multi-dimensional scale of perceived social support Span J Psychol 2012 15 1503 1509 23156952 10.5209/rev_sjop.2012.v15.n3.39434

[B18] Ng CG Amer Siddiq AN Aida SA Zainal NZ Koh OH Validation of the malay version of the multidimensional scale of perceived social support (MSPSS-M) among a group of medical students in faculty of medicine, University Malaya Asian J Psychiatr 2010 3 3 6 23051129 10.1016/j.ajp.2009.12.001

[B19] Guan NC Sulaiman AR Seng LH Ann AYH Wahab S Pillai SK Factorial validity and reliability of the tamil version of multidimensional scale of perceived social support among a group of participants in University Malaya Medical Centre Indian J Psychol Med 2013 35 385 24379500 10.4103/0253-7176.122234 PMC3868091

[B20] Akhtar A Rahman A Husain M Chaudhry IB Duddu V Husain N Multidimensional scale of perceived social support: Psychometric properties in a South Asian population J Obstet Gynaecol Res 2010 36 845 851 20666955 10.1111/j.1447-0756.2010.01204.x

[B21] Nakigudde J Musisi S Ehnvall A Airaksinen E Agren H Adaptation of the multidimensional scale of perceived social support in a Ugandan setting Afr Health Sci 2009 9 Suppl 1 S35 S41 20589159 PMC2890995

[B22] Qadir F Khalid A Haqqani S Medhin G The association of marital relationship and perceived social support with mental health of women in Pakistan BMC Public Health 2013 13 1150 25226599 10.1186/1471-2458-13-1150 PMC3890521

[B23] Cheng S-T Chan ACM The multidimensional scale of perceived social support: dimensionality and age and gender differences in adolescents Pers Indiv Differ 2004 37 1359 1369

[B24] Duru E Re-examination of the psychometric characteristics of the multidimensional scale of perceived social support among Turkish university students Soc Behav Pers Int J 2007 35 443 452

[B25] Golbasi Z Kelleci M Kisacik G Cetin A Prevalence and correlates of depression in pregnancy among Turkish women Matern Child Health J 2010 14 485 491 19238527 10.1007/s10995-009-0459-0

[B26] Yağmur Y Ulukoca N Social support and postpartum depression in low-socioeconomic level postpartum women in Eastern Turkey Int J Public Health 2010 55 543 549 20725761 10.1007/s00038-010-0182-z

[B27] Ege E Timur S Zincir H Geçkil E Sunar-Reeder B Social support and symptoms of postpartum depression among new mothers in Eastern Turkey J Obstet Gynaecol Res 2008 34 585 593 18937713 10.1111/j.1447-0756.2008.00718.x

[B28] Husain N Bevc I Husain M Chaudhry IB Atif N Rahman A Prevalence and social correlates of postnatal depression in a low income country Arch Womens Ment Health 2006 9 197 202 16633740 10.1007/s00737-006-0129-9

[B29] Husain N Cruickshank K Husain M Khan S Tomenson B Rahman A Social stress and depression during pregnancy and in the postnatal period in British Pakistani mothers: a cohort study J Affect Disord 2012 140 268 276 22608713 10.1016/j.jad.2012.02.009 PMC3657151

[B30] Cohen S Wills TA Stress, social support, and the buffering hypothesis Psychol Bull 1985 98 310 3901065

[B31] Kawachi I Berkman LF Social ties and mental health J Urban Health 2001 78 458 467 11564849 10.1093/jurban/78.3.458 PMC3455910

[B32] Beydoun HA Beydoun MA Kaufman JS Lo B Zonderman AB Intimate partner violence against adult women and its association with major depressive disorder, depressive symptoms and postpartum depression: a systematic review and meta-analysis Soc Sci Med 2012 75 959 975 22694991 10.1016/j.socscimed.2012.04.025 PMC3537499

[B33] Kaslow NJ Thompson MP Meadows LA Jacobs D Chance S Gibb B Bornstein H Hollins L Rashid A Phillips K Factors that mediate and moderate the link between partner abuse and suicidal behavior in African American women J Consult Clin Psychol 1998 66 533 540 9642892 10.1037//0022-006x.66.3.533

[B34] Babcock JC Roseman A Green CE Ross JM Intimate partner abuse and PTSD symptomatology: examining mediators and moderators of the abuse-trauma link J Fam Psychol 2008 22 809 818 19102602 10.1037/a0013808

[B35] Coker AL Smith PH Thompson MP McKeown RE Bethea L Davis KE Social support protects against the negative effects of partner violence on mental health J Womens Health Gend Based Med 2002 11 465 476 12165164 10.1089/15246090260137644

[B36] Coker AL Watkins KW Smith PH Brandt HM Social support reduces the impact of partner violence on health: application of structural equation models Prev Med 2003 37 259 267 12914832 10.1016/s0091-7435(03)00122-1

[B37] Stewart RC Umar E Tomenson B Creed F Validation of screening tools for antenatal depression in Malawi-A comparison of the Edinburgh Postnatal Depression Scale and Self Reporting Questionnaire J Affect Disord 2013 150 1041 1047 23769290 10.1016/j.jad.2013.05.036

[B38] First M Spitzer R Williams J Structured Clinical Interview for DSM-IV-TR Axis I Disorders, Research Version, Non-Patient Edition. (SCID-I/NP) New York: Biometrics Research, New York State Psychiatric Institute, November 2002

[B39] Stewart RC Umar E Tomenson B Creed F A cross-sectional study of antenatal depression and associated factors in Malawi Arch Womens Ment Health 2014 17 2 145 154 24240635 10.1007/s00737-013-0387-2

[B40] National Statistics Office Population and Housing Census 2008 Malawi: Zomba Available from: http://unstats.un.org/unsd/demographic/sources/census/2010_PHC/Malawi/Malawi_Report.pdf

[B41] National Statistical Office (NSO) and ICF Macro Malawi Demographic and Health Survey 2010 2011 Zomba, Malawi, and Calverton, Maryland, USA: NSO and ICF Macro 1 603

[B42] Rahman A Iqbal Z Waheed W Hussain N Translation and cultural adaptation of health questionnaires J Pak Med Assoc 2003 53 142 147 12776898

[B43] Stewart RC Kauye F Umar E Vokhiwa M Bunn J Fitzgerald M Tomenson B Rahman A Creed F Validation of a Chichewa version of the self-reporting questionnaire (SRQ) as a brief screening measure for maternal depressive disorder in Malawi, Africa J Affect Disord 2009 112 126 134 18504058 10.1016/j.jad.2008.04.001

[B44] WHO A User’s Guide To The Self Reporting Questionnaire (SRQ) 1994 Geneva: World Health Organisation

[B45] Cox JL Holden JM Sagovsky R Detection of postnatal depression. Development of the 10-item Edinburgh postnatal depression scale Br J Psychiatry 1987 150 782 786 3651732 10.1192/bjp.150.6.782

[B46] Gibson J McKenzie-McHarg K Shakespeare J Price J Gray R A systematic review of studies validating the Edinburgh postnatal depression scale in antepartum and postpartum women Acta Psychiatr Scand 2009 119 350 364 19298573 10.1111/j.1600-0447.2009.01363.x

[B47] National Statistical Office (NSO) [Malawi], and ORC Macro Malawi Demographic and Health Survey 2004 2005 Calverton, Maryland, USA: NSO and ORC Macro

[B48] Hu L Bentler PM Cutoff criteria for fit indexes in covariance structure analysis: Conventional criteria versus new alternatives Struct Equat Model 1999 6 1 1 55

[B49] Pickles A Dunn G Vazquez-Barquero JL Screening for stratification in two-phase (“two- stage”) epidemiological surveys Stat Methods Med Res 1995 4 73 89 7613639 10.1177/096228029500400106

[B50] Allison PD Missing Data. SAGE University Papers Series on Quantitative Applications in the Social Sciences. 07-136 2001 Sage: Thousand Oaks, Ca

[B51] Brown GW Harris T Social Origins of Depression 1978 London: Tavistock 10.1017/s0033291700018791 724871

[B52] Lancaster CA Gold KJ Flynn HA Yoo H Marcus SM Davis MM Risk factors for depressive symptoms during pregnancy: a systematic review Am J Obstet Gynecol 2010 202 5 14 20096252 10.1016/j.ajog.2009.09.007 PMC2919747

[B53] Adewuya AO Ola BA Aloba OO Dada AO Fasoto OO Prevalence and correlates of depression in late pregnancy among Nigerian women Depress Anxiety 2006 24 15 21 16845663 10.1002/da.20221

[B54] Dahlem NW Zimet GD Walker RR The multidimensional scale of perceived social support: a confirmation study J Clin Psychol 1991 47 756 761 1757578 10.1002/1097-4679(199111)47:6<756::aid-jclp2270470605>3.0.co;2-l

[B55] Rahman A Malik A Sikander S Roberts C Creed F Cognitive behaviour therapy-based intervention by community health workers for mothers with depression and their infants in rural Pakistan: a cluster-randomised controlled trial Lancet 2008 372 902 909 18790313 10.1016/S0140-6736(08)61400-2 PMC2603063

